# Efficacy and safety of adjunctive perampanel in patients with focal seizures or generalized tonic‐clonic seizures: Post hoc analysis of Phase II and Phase III double‐blind and open‐label extension studies in India

**DOI:** 10.1002/epi4.12448

**Published:** 2021-02-08

**Authors:** Man M. Mehndiratta, Manoj Gulhane, Shaik A. Jabeen, Anna Patten, Amitabh Dash, Manoj Malhotra

**Affiliations:** ^1^ Janakpuri Super Specialty Hospital Society Janakpuri New Delhi India; ^2^ Curie Manavata Cancer Centre Nashik India; ^3^ Nizam Institute of Medical Science Hyderabad India; ^4^ Eisai Ltd. Hatfield, Hertfordshire UK; ^5^ Eisai Singapore Pte., Ltd. Singapore; ^6^ Eisai Inc. Woodcliff Lake NJ USA

**Keywords:** focal seizures, focal to bilateral tonic‐clonic seizures, generalized tonic‐clonic seizures, perampanel, seizure freedom

## Abstract

**Objective:**

This post hoc analysis assessed the efficacy and safety of adjunctive perampanel in patients (aged ≥ 12 years) with focal seizures (FS), with/without focal to bilateral tonic‐clonic seizures (FBTCS), or generalized tonic‐clonic seizures (GTCS) in India.

**Methods:**

Centers in India were identified from six double‐blind, randomized, Phase II and Phase III studies of adjunctive perampanel (2–12 mg/day) and their open‐label extensions (OLEx). Efficacy assessments included median percent change in seizure frequency per 28 days, 50% and 75% responder and seizure‐freedom rates. Treatment‐emergent adverse events (TEAEs) were monitored.

**Results:**

Overall, 128 patients (placebo, n = 39; perampanel, n = 89) were included in the double‐blind Safety Analysis Set and 126 (FS, n = 113 [placebo, n = 32; perampanel, n = 81]; FBTCS, n = 35 [placebo, n = 14; perampanel, n = 21]; GTCS, n = 13 [placebo, n = 6; perampanel, n = 7]) comprised the Full Analysis Set. Median percent reductions in seizure frequency per 28 days for placebo vs perampanel for Indian patients were as follows: 34.8% vs 49.8% (FS; not significant [NS]) and 43.1% vs 60.5% (FBTCS; NS) at 4–12 mg/day, respectively, and −22.4% vs 8.2% (GTCS; NS) at 8 mg/day, respectively. Fifty‐percent responder rates were 37.5% vs 55.1% (FS; NS), 42.9% vs 60.0% (FBTCS; NS), and 16.7% vs 42.9% (GTCS; NS), respectively; seizure‐freedom rates were 0.0% vs 5.8%, 7.1% vs 10.0%, and 0.0% vs 14.3%, respectively (all NS). Overall, 110 patients entered OLEx studies (FS, n = 99; GTCS, n = 11). Perampanel was efficacious for up to four years for FS and FBTCS and two years for GTCS. Across double‐blind and OLEx studies, TEAEs occurred in 58.4% and 83.6% of Indian perampanel‐treated patients, respectively; dizziness was most common. Efficacy and safety outcomes were generally similar overall between Indian and non‐Indian patients.

**Significance:**

These data suggest adjunctive perampanel (up to 12 mg/day) may be a suitable anti‐seizure medication for patients (aged ≥ 12 years) with FS, with/without FBTCS, or GTCS in India.

**Key Points**

- This post hoc analysis assessed efficacy and safety of adjunctive perampanel in patients with epilepsy from India and non‐Indian patients
- Adjunctive perampanel conferred greater reductions in seizure frequency vs placebo for focal and generalized tonic‐clonic seizures (GTCS)
- Perampanel showed efficacy for up to four years for focal seizures and two years for GTCS in Indian and non‐Indian patients
- Long‐term adjunctive perampanel treatment was well tolerated in Indian patients; the safety profile was similar with non‐Indian patients
- Adjunctive perampanel may be a suitable treatment in patients from India with focal seizures or GTCS

## 1 INTRODUCTION

Epilepsy affects approximately 50 million people worldwide^1^ and is associated with high economic, social, and psychological burden, particularly in Asia where discrimination against people with epilepsy is more common than in other countries.^1^, ^2^ Approximately 23 million people in Asia have epilepsy,^3^ with variable prevalence and incidence rates among Asian countries.^3^ In India, prevalence and incidence rates are estimated as 3.0–11.9 in 1000 and 38.0–60.0 in 100 000, respectively, whereas in China these are 4.6–7.0 in 1000 and 28.8–35.0 in 100 000, respectively.^3^, ^4^, ^5^

Anti‐seizure medications (ASMs) are the primary treatment for epilepsy, yet access varies across Asia; newer ASMs with more favorable safety profiles may not be available in all countries.^2^ New ASMs are often studied in select groups of patients in Europe and the USA^2^; however, results in these populations do not always translate to Asian populations, given differences in genetic backgrounds,^2^ which may cause variabilities in drug response and dosing recommendations.^6^ Since licensing of new drugs in Asia is by individual countries,^3^ it is important to assess ASM efficacy and safety at the individual country level.

Perampanel, an orally active, non‐competitive, selective α‐amino‐3‐hydroxy‐5‐methyl‐4‐isoxazolepropionic acid (AMPA) receptor antagonist,^7^, ^8^ is approved as adjunctive therapy for focal seizures (FS; previously partial‐onset seizures), with or without focal to bilateral tonic‐clonic seizures (FBTCS; previously secondarily generalized seizures), in patients aged ≥ 12 years in >55 countries, and generalized tonic‐clonic seizures (GTCS; previously primary generalized tonic‐clonic seizures) in patients aged ≥ 12 years in >50 countries (data on file, Eisai Inc., Woodcliff Lake, NJ, USA). In India, perampanel is approved for adjunctive treatment of FS, with or without FBTCS, and GTCS in patients with epilepsy aged ≥ 12 years (data on file, Eisai Inc., Woodcliff Lake, NJ, USA).^9^

Clinical development of adjunctive perampanel included a number of Phase II and Phase III, double‐blind, randomized, placebo‐controlled studies in patients aged ≥ 12 years with FS, with or without FBTCS: international Phase II Study 235 (NCT01161524); international Phase III Studies 304 (NCT00699972), 305 (NCT00699582), and 306 (NCT00700310); and Asia‐Pacific Phase III Study 335 (NCT01618695)^10^, ^11^, ^12^, ^13^, ^14^; or idiopathic generalized epilepsy and GTCS in international Phase III Study 332 (NCT01393743).^15^ Since randomized trials offer relatively short exposures to investigational ASMs (~8–12 weeks), longer‐term follow‐up studies are important to assess efficacy and adverse side effects that may only occur after long‐term exposure.^16^ To assess long‐term efficacy and safety of adjunctive perampanel, eligible patients who completed the double‐blind studies could enter open‐label extension (OLEx) studies: Study 235 OLEx (NCT01161524),^17^ OLEx Study 307 (NCT00735397; Studies 304, 305, and 306),^18^ Study 335 OLEx (NCT01618695; data on file, Eisai Co., Ltd., Tokyo, Japan), and Study 332 OLEx (NCT01393743; data on file, Eisai Inc., Woodcliff Lake, NJ, USA).

We report results of a post hoc analysis performed using data from study centers in India and remaining centers (non‐Indian) from the double‐blind and OLEx studies to assess the efficacy and safety of adjunctive perampanel in Indian patients (aged ≥ 12 years) with FS (with or without FBTCS) or GTCS.

## 2 METHODS

### 2.1 Study designs

For this post hoc analysis, Studies 235, 305, 306, and 332 were identified as recruiting patients from centers in India (Study 235, n = 8 sites; Study 305, n = 4 sites; Study 306, n = 8 sites; Study 332, n = 4 sites). The designs of the double‐blind studies have been previously reported.^10^, ^11^, ^12^, ^13^, ^14^, ^15^ Briefly, patients aged ≥ 12 years with uncontrolled FS (with or without FBTCS) or GTCS, despite treatment with 1–3 ASMs, were randomized to once‐daily placebo or adjunctive perampanel 2–12 mg/day (dependent on study), across a double‐blind treatment phase. Studies 235, 304, 305, 306, and 335 included a 19‐week double‐blind treatment phase (6‐week titration; 13‐week maintenance); Study 332 included a 17‐week double‐blind treatment phase (4‐week titration; 13‐week maintenance). Most studies used 1:1 randomization for placebo vs perampanel; however, Study 235 used 1:2 randomization.

OLEx Studies 235 OLEx, 307, and 332 OLEx included patients from centers in India. All OLEx studies comprised a blinded conversion period (6–16 weeks across studies), where perampanel dose optimization was achieved (maximum 12 mg/day). This was followed by a 27‐ to 256‐week open‐label maintenance period (Study 235 OLEx: 27 weeks^17^; Study 307: 256 weeks^18^; Study 335 OLEx: ≥46 weeks [data on file, Eisai Co., Ltd., Tokyo, Japan]; Study 332 OLEx: 136 weeks [data on file, Eisai Inc., Woodcliff Lake, NJ, USA]), resulting in up to 1‐ to 5‐year perampanel exposure across studies. Patients who previously received placebo during the double‐blind studies were blindly converted to adjunctive perampanel during the OLEx conversion period; patients who previously received perampanel during the double‐blind studies continued during the OLEx studies. During the conversion period, patients had their perampanel dose up‐titrated in 2‐mg increments weekly (Studies 235 OLEx, 335 OLEx, and 332 OLEx) or every 2 weeks (Study 307) from the dose on which they completed the double‐blind study (or from 2 mg for patients previously receiving placebo), to a maximum of 12 mg/day, based on tolerability and seizure control. During the maintenance period, patients were unblinded to study treatment and remained on the optimal perampanel dose established during the blinded conversion period. Adjustment of perampanel dose during the maintenance period was permitted at the investigator's discretion, based on tolerability.

All studies were performed in accordance with the relevant Good Clinical Practice Guidelines. Trial protocols, amendments, and informed consent were reviewed by national regulatory authorities and independent ethics committees or institutional review boards. All patients gave written informed consent before participation.^10^, ^11^, ^12^, ^13^, ^14^, ^15^, ^17^, ^18^

### 2.2 Post hoc efficacy assessments

Efficacy assessments were based on the Full Analysis Set and split by seizure type (FS, FBTCS, GTCS). For the double‐blind study analyses, the Full Analysis Set comprised all patients who received ≥1 dose of study drug (placebo or perampanel) and had any seizure frequency data during the double‐blind treatment phase. For the OLEx study analyses, the Full Analysis Set comprised all patients who received ≥1 dose of perampanel during the OLEx study and had baseline seizure frequency data and any valid seizure data during perampanel treatment (defined below).

Efficacy assessments for up to four years (FS and FBTCS) or up to two years (GTCS) included the following: median percent change in seizure frequency per 28 days relative to double‐blind or pre‐perampanel baseline (defined below); 50% and 75% responder rates (defined as the proportion of patients with a ≥50% or ≥75% reduction in seizure frequency per 28 days during the double‐blind study maintenance period or each respective year of the perampanel treatment duration; last observation carried forward [LOCF]); and seizure‐freedom rates. For the double‐blind study analyses, seizure freedom was defined as the proportion of patients who were study completers and had no seizures during the double‐blind maintenance period; for the OLEx study analyses, this was the proportion of patients who were free from seizures during that period of the perampanel treatment duration.

During the double‐blind studies, all efficacy assessments were performed for placebo vs perampanel 2, 4, 8, and 12 mg/day and 4–12 mg/day combined for FS and FBTCS, and for placebo vs perampanel 8 mg/day for GTCS. During OLEx studies, all patients received perampanel; therefore, no placebo comparison was included.

### 2.3 Post hoc safety assessments

Safety assessments were based on the Safety Analysis Set. For the double‐blind study analyses, the Safety Analysis Set comprised all patients who received ≥1 dose of study drug and had ≥1 post‐dose safety assessment. For the OLEx study analyses, the Safety Analysis Set comprised all patients who received ≥1 dose of perampanel during the OLEx study and had any on‐treatment safety data during the OLEx study.

Safety data were pooled for all seizure types and analyses included monitoring of treatment‐emergent adverse events (TEAEs), serious TEAEs, and TEAEs leading to withdrawal. A TEAE was defined as an adverse event with an onset date, or worsening in severity from baseline (pre‐treatment), on or after the first dose of study drug up to 30 days following study‐drug discontinuation. Treatment‐related TEAEs were those that were considered to be possibly or probably related to study treatment by the investigator.

### 2.4 Statistical analyses

Perampanel treatment duration started from the first dose of perampanel in the double‐blind study to the last dose of perampanel in the OLEx period, except for patients who had a gap in perampanel exposure from the double‐blind study to the OLEx period of >14 days; for these patients, the perampanel treatment duration was the OLEx exposure.

For patients who received placebo during the double‐blind studies, pre‐perampanel baseline included seizure diary data collected during the double‐blind study. For patients who received perampanel during the double‐blind studies, pre‐perampanel baseline included seizure diary data collected during the baseline period (pre‐randomization monitoring phase) of the double‐blind study plus four weeks prior.

For the double‐blind study analyses, median difference to placebo and 95% confidence intervals (CIs) was based on the Hodges‐Lehmann estimator. *P*‐values for median percent change were based on a rank analysis of covariance with treatment as a factor and pre‐randomization seizure frequency as a covariate, and for responder/seizure‐freedom rates were based on non‐missing values from a Cochran‐Mantel‐Haenszel test.

For OLEx analyses and to account for patients who dropped out of the study early, sensitivity analyses were conducted for efficacy assessments. For these, the LOCF approach was used, meaning that patients who completed or withdrew from the study had their last year of treatment carried forward to later time points; for patients who were treated for <1 year, their entire treatment period was carried forward to later time points.

## 3 RESULTS

### 3.1 Double‐blind studies: patients

Across double‐blind studies, 128 patients were identified from centers in India and included in the pooled Safety Analysis Set (placebo, n = 39; perampanel, n = 89). Patient demographics and baseline characteristics in the Indian population were generally similar between the placebo and perampanel groups, except for sex in which there was a higher proportion of female patients in the perampanel group compared with the placebo group (Table 1). The mean age and median body mass index (BMI) of the non‐Indian population were slightly higher than the Indian population, and the proportion of female patients was more evenly distributed across treatment groups in the non‐Indian population (Table 1). The most common seizure type during baseline in the placebo and perampanel groups was focal impaired awareness seizures (Indian: 43.6% and 61.8%, respectively; non‐Indian: 72.9% and 74.5%, respectively).

**TABLE 1:** Indian and non‐Indian patient demographics and baseline characteristics during the double‐blind and OLEx studies (Safety Analysis Set)

	Double‐blind studies						OLEx studies
	Placebo	Perampanel					Perampanel
		2 mg/day	4 mg/day	8 mg/day	12 mg/day	Total	
Indian patients							
n	39	12	9	54	14	89	110
Mean age, y (SD)	22.0 (8.9)	24.8 (8.5)	29.3 (6.8)	20.2 (8.3)	29.7 (11.2)	23.2 (9.5)	23.1 (9.2)
Female, n (%)	10 (25.6)	8 (66.7)	2 (22.2)	21 (38.9)	6 (42.9)	37 (41.6)	41 (37.3)
Median BMI, kg/m^2^ (min, max)	20.6 (14.2, 33.8)	18.6 (16.4, 25.7)	21.6 (17.5, 28.5)	18.4 (15.4, 33.6)	21.5 (15.9, 36.7)	19.2 (15.4, 36.7)	20.1 (14.2, 33.8)
Seizure type, n (%)							
Focal aware without motor signs	2 (5.1)	1 (8.3)	0 (0.0)	0 (0.0)	0 (0.0)	1 (1.1)	3 (2.7)
Focal aware with motor signs	11 (28.2)	4 (33.3)	2 (22.2)	7 (13.0)	3 (21.4)	16 (18.0)	23 (20.9)
Focal impaired awareness	17 (43.6)	9 (75.0)	5 (55.6)	33 (61.1)	8 (57.1)	55 (61.8)	62 (56.4)
Focal with FBTCS	15 (38.5)	1 (8.3)	4 (44.4)	11 (20.4)	5 (35.7)	21 (23.6)	29 (26.4)
Tonic‐clonic	6 (15.4)	0 (0.0)	0 (0.0)	7 (13.0)	0 (0.0)	7 (7.9)	11 (10.0)
Myoclonic	0 (0.0)	0 (0.0)	0 (0.0)	1 (1.9)	0 (0.0)	1 (1.1)	1 (0.9)
Absence	0 (0.0)	0 (0.0)	0 (0.0)	0 (0.0)	0 (0.0)	0 (0.0	0 (0.0)
Non‐Indian patients							
n	709	168	339	718	421	1646	2037
Mean age, y (SD)	33.1 (13.8)	34.5 (13.7)	33.5 (12.8)	32.8 (14.3)	34.7 (13.7)	33.6 (13.8)	33.0 (13.6)
Female, n (%)	368 (51.9)	87 (51.8)	176 (51.9)	366 (51.0)	223 (53.0)	852 (51.8)	38 (51.0)
Median BMI, kg/m^2^ (min, max)	23.5 (12.3, 50.5)	23.6 (16.4, 41.7)	23.4 (11.9, 39.6)	23.7 (14.1, 47.4)	23.5 (11.1, 45.7)	23.6 (11.1, 47.4)	23.4 (11.1, 50.5)
Seizure type, n (%)							
Focal aware without motor signs	137 (19.3)	34 (20.2)	62 (18.3)	151 (21.0)	85 (20.2)	332 (20.2)	388 (19.0)
Focal aware with motor signs	157 (22.1)	41 (24.4)	100 (29.5)	176 (24.5)	98 (23.3)	415 (25.2)	492 (24.2)
Focal impaired awareness	517 (72.9)	133 (79.2)	259 (76.4)	497 (69.2)	338 (80.3)	227 (74.5)	513 (74.3)
Focal with FBTCS	232 (32.7)	67 (39.9)	125 (36.9)	229 (31.9)	158 (37.5)	579 (35.2)	709 (34.8)
Tonic‐clonic	76 (10.7)	0 (0.0)	0 (0.0)	74 (10.3)	0 (0.0)	74 (4.5)	127 (6.2)
Myoclonic	24 (3.4)	0 (0.0)	0 (0.0)	23 (3.2)	0 (0.0)	23 (1.4)	43 (2.1)
Absence	33 (4.7)	0 (0.0)	0 (0.0)	27 (3.8)	0 (0.0)	27 (1.6)	50 (2.5)
Tonic	2 (0.3)	0 (0.0)	0 (0.0)	0 (0.0)	0 (0.0)	0 (0.0)	2 (0.1)
Atonic	1 (0.1)	0 (0.0)	0 (0.0)	0 (0.0)	0 (0.0)	0 (0.0)	0 (0.0)

Abbreviations: BMI, body mass index; FBTCS, focal to bilateral tonic‐clonic seizures; max, maximum; min, minimum; OLEx, open‐label extension; SD, standard deviation.

Most Indian patients were receiving one or two concomitant ASMs during baseline (placebo, n = 11 [28.2%] and n = 21 [53.8%]; perampanel, n = 25 [28.1%] and n = 44 [49.4%], respectively); 29 (74.4%) placebo‐treated patients and 65 (73.0%) perampanel‐treated patients were receiving an enzyme‐inducing ASM (EIASM; carbamazepine, oxcarbazepine, phenytoin, and eslicarbazepine). The most common non‐EIASMs during baseline were valproic acid (placebo, n = 17 [43.6%]; perampanel, n = 26 [29.2%]), clobazam (placebo, n = 12 [30.8%]; perampanel, n = 25 [28.1%]), and levetiracetam (placebo, n = 4 [10.3%]; perampanel, n = 27 [30.3%]). The most common EIASM was carbamazepine (placebo, n = 15 [38.5%]; perampanel, n = 40 [44.9%]).

The Full Analysis Set included 126 Indian patients. Of these, 113 patients (placebo, n = 32; perampanel, n = 81) had FS, of which 35 had FBTCS during baseline (placebo, n = 14; perampanel, n = 21), and 13 patients (placebo, n = 6; perampanel, n = 7) had GTCS. Median (minimum, maximum) baseline seizure frequency per 28 days for placebo and perampanel 2, 4, 8, and 12 mg/day was: 6.6 (0.8, 169.4), 10.0 (4.1, 133.3), 4.9 (2.9, 102.4), 5.0 (0.8, 89.5), and 10.5 (3.8, 30.7) for FS, respectively; and 4.1 (0.7, 169.4), 1.2 (1.2, 1.2), 3.8 (1.1, 9.6), 2.4 (0.7, 12.4), and 6.4 (1.4, 19.1) for FBTCS, respectively. For GTCS, median (minimum, maximum) baseline seizure frequency per 28 days was 3.4 (1.4, 4.5) for placebo and 2.4 (2.0, 9.8) for perampanel 8 mg/day.

### 3.2 Double‐blind studies: efficacy outcomes

**FIGURE 1:**
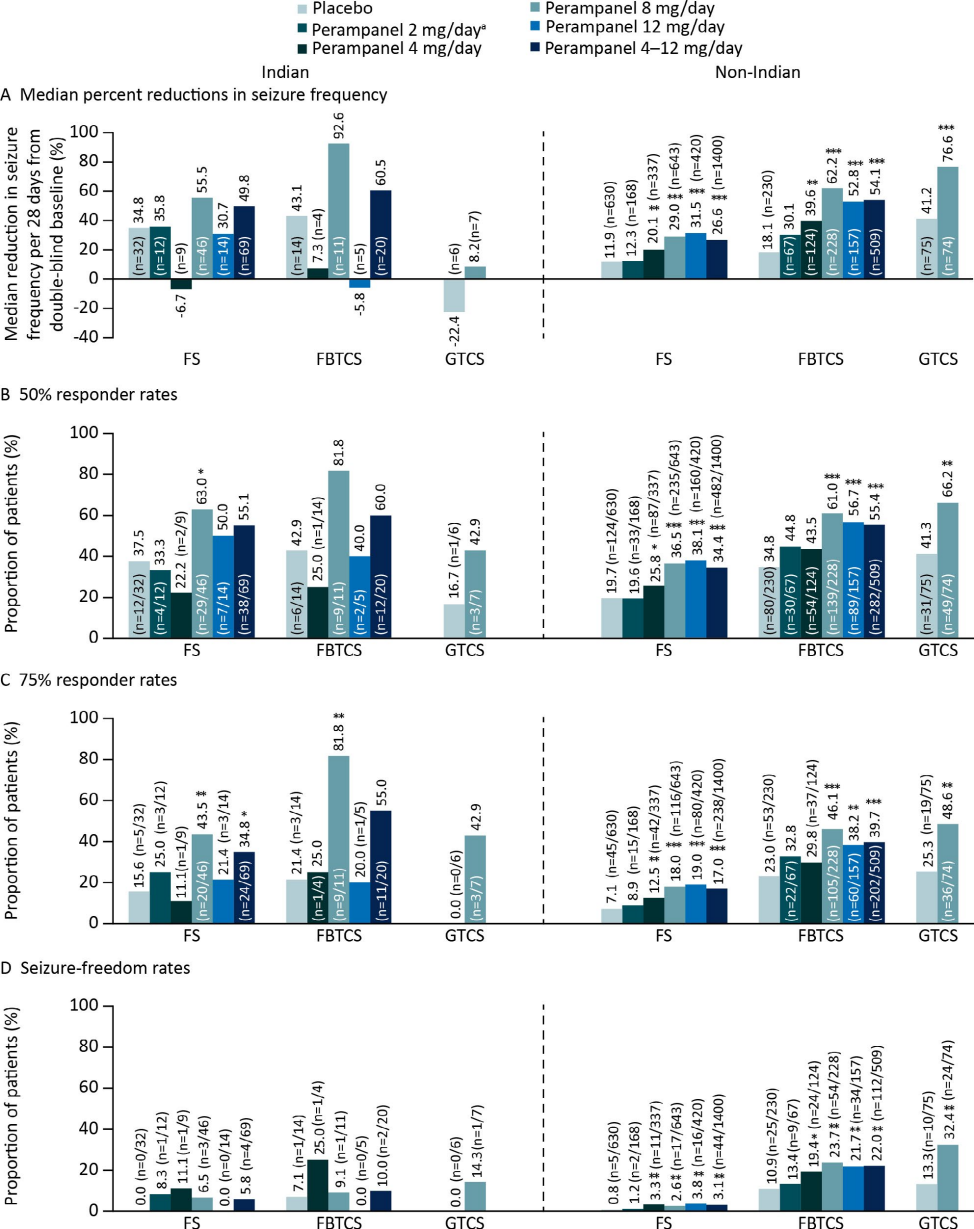
Double‐blind studies: A, Median percent reductions in seizure frequency per 28 days from baseline; B, 50% responder rates; C, 75% responder rates, and D, Seizure‐freedom rates during maintenance for Indian and non‐Indian patients (Full Analysis Set). Abbreviations: FBTCS, focal to bilateral tonic‐clonic seizures; FS, focal seizures; GTCS, generalized tonic‐clonic seizures. *P ≤ .05, **P ≤ .01, ***P < .0001 vs placebo. ^a^Data not shown for perampanel 2 mg/day in Indian patients with FBTCS due to small patient number (n = 1)

Median percent reductions in seizure frequency per 28 days for each seizure type are shown in Figure 1A for Indian and non‐Indian patients. For FS, perampanel 8 and 4–12 mg/day conferred greater median percent reductions in seizure frequency in Indian patients vs placebo; however, statistical significance was not reached. For FBTCS, median percent reductions in seizure frequency were greater with perampanel 8 and 4–12 mg/day vs placebo, but again, statistical significance was not observed. In non‐Indian patients, all perampanel doses (except 2 mg/day) conferred significantly greater reductions in seizure frequency for FS and FBTCS vs placebo. For GTCS, perampanel 8 mg/day conferred greater median percent reductions in seizure frequency vs placebo in both Indian (not significant) and non‐Indian (*P *< .0001) patients. The smaller sample size for the Indian cohort should be considered when interpreting these data. Median (95% CI) differences for perampanel vs placebo for each seizure type and cohort are provided in Table S1.

Responder and seizure‐freedom rates for Indian and non‐Indian patients are shown in Figures 1B‐D. In Indian patients, perampanel 4–12 mg/day was associated with greater 50% responder rates, 75% responder rates, and seizure‐freedom rates for FS and FBTCS vs placebo, although only 75% responder rates for FS reached statistical significance (*P *< .05); in non‐Indian patients, perampanel 4–12 mg/day conferred significant efficacy vs placebo as did perampanel doses of 8 and 12 mg/day for 50%, 75%, and seizure‐ freedom rates. Perampanel 8 mg/day was also associated with greater 50% and 75% responder and seizure‐ freedom rates vs placebo for GTCS in Indian (not significant) and non‐Indian patients (*P *≤ .01).

### 3.3 Double‐blind studies: safety outcomes

TEAEs were reported in 21 (53.8%) and 52 (58.4%) placebo‐ and perampanel‐treated Indian patients, respectively (Table 2), compared with 480 (67.7%) and 1288 (78.3%) non‐Indian patients (Table 3). Treatment‐related TEAEs (those considered possibly or probably related to study treatment) were reported in 15 (38.5%) and 35 (39.3%) placebo‐ and perampanel‐treated Indian patients compared with 276 (38.9%) and 1015 (61.7%) non‐Indian patients, respectively. The most common TEAEs with perampanel were dizziness, headache, and pyrexia in Indian patients (Table 2), and dizziness, somnolence, and headache in non‐Indian patients (Table 3). There were no deaths reported of Indian patients; however, one (2.6%) placebo‐treated patient and two (2.2%) perampanel‐treated patients experienced serious TEAEs (placebo: convulsion; perampanel: ankle fracture and delirium [both 2 mg/day]). The serious TEAE of delirium with perampanel 2 mg/day led to discontinuation (this patient also had a non‐serious TEAE of blood sodium decreased). There were no other serious TEAEs that led to discontinuation during the double‐blind studies in Indian patients. In comparison, two (0.1%) non‐Indian patients died (sudden cardiac death in a Chinese patient receiving placebo and unknown cause in a Korean patient receiving perampanel; both events were considered unrelated to study treatment), and there were 40 (5.6%) and 91 (5.5%) serious TEAEs in placebo‐ and perampanel‐treated patients respectively. TEAEs leading to discontinuation were reported in 33 (4.7%) placebo‐treated and 165 (10.0%) perampanel‐treated non‐Indian patients.

**TABLE 2:** Overview of TEAEs and most common TEAEs occurring in ≥4% of Indian patients in the total perampanel group during the double‐blind or OLEx studies (Safety Analysis Set)

	Double‐blind studies						OLEx studies
	Placebo (n = 39)	Perampanel					Perampanel (n = 110)
		2 mg/day (n = 12)	4 mg/day (n = 9)	8 mg/day (n = 54)	12 mg/day (n = 14)	Total (n = 89)	
TEAEs, n (%)	21 (53.8)	7 (58.3)	2 (22.2)	35 (64.8)	8 (57.1)	52 (58.4)	92 (83.6)
Treatment‐related TEAEs, n (%)	15 (38.5)	3 (25.0)	1 (11.1)	25 (46.3)	6 (42.9)	35 (39.3)	65 (59.1)
Serious TEAEs, n (%)	1 (2.6)	2 (16.7)	0 (0.0)	0 (0.0)	0 (0.0)	2 (2.2)	9 (8.2)
TEAEs leading to study‐drug withdrawal, n (%)	0 (0.0)	1 (8.3)	0 (0.0)	0 (0.0)	0 (0.0)	1 (1.1)	8 (7.3)
Most common (≥4%) TEAEs, n (%)							
Dizziness	2 (5.1)	0 (0.0)	0 (0.0)	7 (13.0)	3 (21.4)	10 (11.2)	23 (20.9)
Pyrexia	2 (5.1)	3 (25.0)	0 (0.0)	5 (9.3)	0 (0.0)	8 (9.0)	24 (21.8)
Headache	3 (7.7)	2 (16.7)	0 (0.0)	5 (9.3)	1 (7.1)	8 (9.0)	15 (13.6)
Somnolence	0 (0.0)	1 (8.3)	0 (0.0)	5 (9.3)	0 (0.0)	6 (6.7)	16 (14.5)
Eosinophilia	3 (7.7)	0 (0.0)	0 (0.0)	4 (7.4)	0 (0.0)	4 (4.5)	17 (15.5)
Weight increased	1 (2.6)	0 (0.0)	0 (0.0)	3 (5.6)	1 (7.1)	4 (4.5)	11 (10.0)
Convulsion	3 (7.7)	0 (0.0)	0 (0.0)	1 (1.9)	0 (0.0)	1 (1.1)	11 (10.0)
Decreased appetite	1 (2.6)	0 (0.0)	0 (0.0)	1 (1.9)	2 (14.3)	3 (3.4)	7 (6.4)
Upper respiratory tract infection	0 (0.0)	0 (0.0)	0 (0.0)	2 (3.7)	0 (0.0)	2 (2.2)	10 (9.1)
Aggression	1 (2.6)	0 (0.0)	0 (0.0)	3 (5.6)	0 (0.0)	3 (3.4)	5 (4.5)
Ataxia	0 (0.0)	0 (0.0)	0 (0.0)	1 (1.9)	0 (0.0)	1 (1.1)	10 (9.1)
Nasopharyngitis	0 (0.0)	1 (8.3)	0 (0.0)	1 (1.9)	0 (0.0)	2 (2.2)	8 (7.3)
Nausea	0 (0.0)	0 (0.0)	0 (0.0)	0 (0.0)	2 (14.3)	2 (2.2)	6 (5.5)
Vomiting	1 (2.6)	0 (0.0)	0 (0.0)	2 (3.7)	0 (0.0)	2 (2.2)	5 (4.5)
Gamma‐glutamyltransferase increased	0 (0.0)	0 (0.0)	0 (0.0)	0 (0.0)	1 (7.1)	1 (1.1)	6 (5.5)
Balance disorder	0 (0.0)	0 (0.0)	0 (0.0)	0 (0.0)	1 (7.1)	1 (1.1)	5 (4.5)
Fall	1 (2.6)	0 (0.0)	0 (0.0)	0 (0.0)	0 (0.0)	0 (0.0)	6 (5.5)
Head injury	1 (2.6)	0 (0.0)	0 (0.0)	0 (0.0)	0 (0.0)	0 (0.0)	5 (4.5)
Vertigo	1 (2.6)	0 (0.0)	0 (0.0)	0 (0.0)	0 (0.0)	0 (0.0)	5 (4.5)

Abbreviations: AE, adverse event; OLEx, open‐label extension; TEAE, treatment‐emergent adverse event.

A TEAE is defined as an AE with an onset date, or a worsening in severity from baseline, on or after the first dose of study drug up to 30 days following study‐ drug discontinuation. A patient with ≥2 AEs in the same system organ class or with the same preferred term is counted only once for that system organ class or preferred term.

**TABLE 3:** Overview of TEAEs and most common TEAEs occurring in ≥4% of non‐Indian patients in the total perampanel group during the double‐blind or OLEx studies (Safety Analysis Set)

	Double‐blind studies						OLEx studies
	Placebo (n = 709)	Perampanel					Perampanel (n = 2037)
		2 mg/day (n = 168)	4 mg/day (n = 339)	8 mg/day (n = 718)	12 mg/day (n = 421)	Total (n = 1646)	
TEAEs, n (%)	480 (67.7)	104 (61.9)	230 (67.8)	579 (80.6)	375 (89.1)	1288 (78.3)	1853 (91.0)
Treatment‐related TEAEs, n (%)	276 (38.9)	64 (38.1)	157 (46.3)	470 (65.5)	324 (77.0)	1015 (61.7)	1644 (80.7)
Serious TEAEs, n (%)	40 (5.6)	4 (2.4)	12 (3.5)	42 (5.8)	33 (7.8)	91 (5.5)	385 (18.9)
TEAEs leading to study‐drug withdrawal, n (%)	33 (4.7)	11 (6.5)	13 (3.8)	67 (9.3)	74 (17.6)	165 (10.0)	355 (17.4)
Most common (≥4%) TEAEs, n (%)							
Dizziness	60 (8.5)	18 (10.7)	68 (20.1)	232 (32.3)	182 (43.2)	500 (30.4)	909 (44.6)
Somnolence	60 (8.5)	21 (12.5)	44 (13.0)	115 (16.0)	77 (18.3)	257 (15.6)	429 (21.1)
Headache	75 (10.6)	14 (8.3)	31 (9.1)	76 (10.6)	43 (10.2)	164 (10.0)	315 (15.5)
Nasopharyngitis	54 (7.6)	6 (3.6)	32 (9.4)	57 (7.9)	34 (8.1)	129 (7.8)	288 (14.1)
Fatigue	32 (4.5)	8 (4.8)	17 (5.0)	61 (8.5)	40 (9.5)	126 (7.7)	226 (11.1)
Weight increased	9 (1.3)	3 (1.8)	14 (4.1)	32 (4.5)	16 (3.8)	65 (3.9)	197 (9.7)
Upper respiratory tract infection	28 (3.9)	11 (6.5)	14 (4.1)	34 (4.7)	21 (5.0)	80 (4.9)	160 (7.9)
Irritability	17 (2.4)	7 (4.2)	15 (4.4)	54 (7.5)	39 (9.3)	115 (7.0)	120 (5.9)
Nausea	30 (4.2)	4 (2.4)	9 (2.7)	36 (5.0)	28 (6.7)	77 (4.7)	144 (7.1)
Fall	17 (2.4)	2 (1.2)	3 (0.9)	26 (3.6)	26 (6.2)	57 (3.5)	124 (6.1)
Convulsion	22 (3.1)	3 (1.8)	6 (1.8)	19 (2.6)	11 (2.6)	39 (2.4)	127 (6.2)
Diarrhea	29 (4.1)	2 (1.2)	6 (1.8)	23 (3.2)	16 (3.8)	47 (2.9)	109 (5.4)
Insomnia	29 (4.1)	2 (1.2)	5 (1.5)	23 (3.2)	14 (3.3)	44 (2.7)	111 (5.4)
Vomiting	25 (3.5)	5 (3.0)	4 (1.2)	22 (3.1)	14 (3.3)	45 (2.7)	113 (5.5)
Vertigo	7 (1.0)	6 (3.6)	10 (2.9)	26 (3.6)	16 (3.8)	58 (3.5)	100 (4.9)
Gait disturbance	10 (1.4)	1 (0.6)	4 (1.2)	22 (3.1)	19 (4.5)	46 (2.8)	105 (5.2)
Contusion	14 (2.0)	1 (0.6)	5 (1.5)	22 (3.1)	11 (2.6)	39 (2.4)	102 (5.0)
Ataxia	1 (0.1)	0 (0.0)	3 (0.9)	20 (2.8)	28 (6.7)	51 (3.1)	102 (5.0)
Anxiety	8 (1.1)	4 (2.4)	4 (1.2)	24 (3.3)	10 (2.4)	42 (2.6)	96 (4.7)
Influenza	17 (2.4)	2 (1.2)	6 (1.8)	14 (1.9)	7 (1.7)	29 (1.8)	95 (4.7)
Aggression	2 (0.3)	1 (0.6)	5 (1.5)	17 (2.4)	14 (3.3)	37 (2.2)	98 (4.8)
Back pain	9 (1.3)	1 (0.6)	4 (1.2)	11 (1.5)	14 (3.3)	30 (1.8)	98 (4.8)
Rash	10 (1.4)	2 (1.2)	6 (1.8)	18 (2.5)	14 (3.3)	40 (2.4)	85 (4.2)
Balance disorder	3 (0.4)	0 (0.0)	4 (1.2)	28 (3.9)	11 (2.6)	43 (2.6)	89 (4.4)
Pyrexia	10 (1.4)	3 (1.8)	4 (1.2)	13 (1.8)	7 (1.7)	27 (1.6)	96 (4.7)
Depression	9 (1.3)	1 (0.6)	1 (0.3)	7 (1.0)	8 (1.9)	17 (1.0)	97 (4.8)
Dysarthria	1 (0.1)	0 (0.0)	2 (0.6)	18 (2.5)	13 (3.1)	33 (2.0)	83 (4.1)

Abbreviations: AE, adverse event; OLEx, open‐label extension; TEAE, treatment‐emergent adverse event.

A TEAE is defined as an AE with an onset date, or a worsening in severity from baseline, on or after the first dose of study drug up to 30 days following study‐drug discontinuation. A patient with ≥2 AEs in the same system organ class or with the same preferred term is counted only once for that system organ class or preferred term.

### 3.4 OLEx studies: patients

Overall, 110 patients from India entered the OLEx studies and were included in the Safety Analysis Set. Time to discontinuation is shown in Figure S1. Patient demographics and baseline characteristics were generally similar to those for patients in the double‐blind studies for both the Indian and non‐Indian patients (Table 1). Like the double‐blind studies, mean age and median BMI were higher in the non‐Indian population compared with the Indian population, and there was a higher proportion of female patients in the non‐Indian population compared with the Indian population.

During baseline, 31 (28.2%), 56 (50.9%), and 23 (20.9%) Indian patients were receiving one, two, and three concomitant ASMs, respectively; 83 (75.5%) patients were receiving EIASMs. The most common non‐EIASMs were valproic acid (n = 34 [30.9%]), clobazam (n = 31 [28.2%]), and levetiracetam (n = 25 [22.7%]); the most common EIASM was carbamazepine (n = 50 [45.5%]).

The Full Analysis Set included 110 patients; of these, 99 patients had FS, of which 33 had FBTCS during baseline, and 11 had GTCS. Median (minimum, maximum) pre‐perampanel baseline seizure frequency per 28 days was 6.8 (0.2, 133.3) for FS, 2.8 (0.2, 103.7) for FBTCS, and 2.9 (1.3, 9.8) for GTCS.

### 3.5 OLEx studies: efficacy outcomes

**FIGURE 2:**
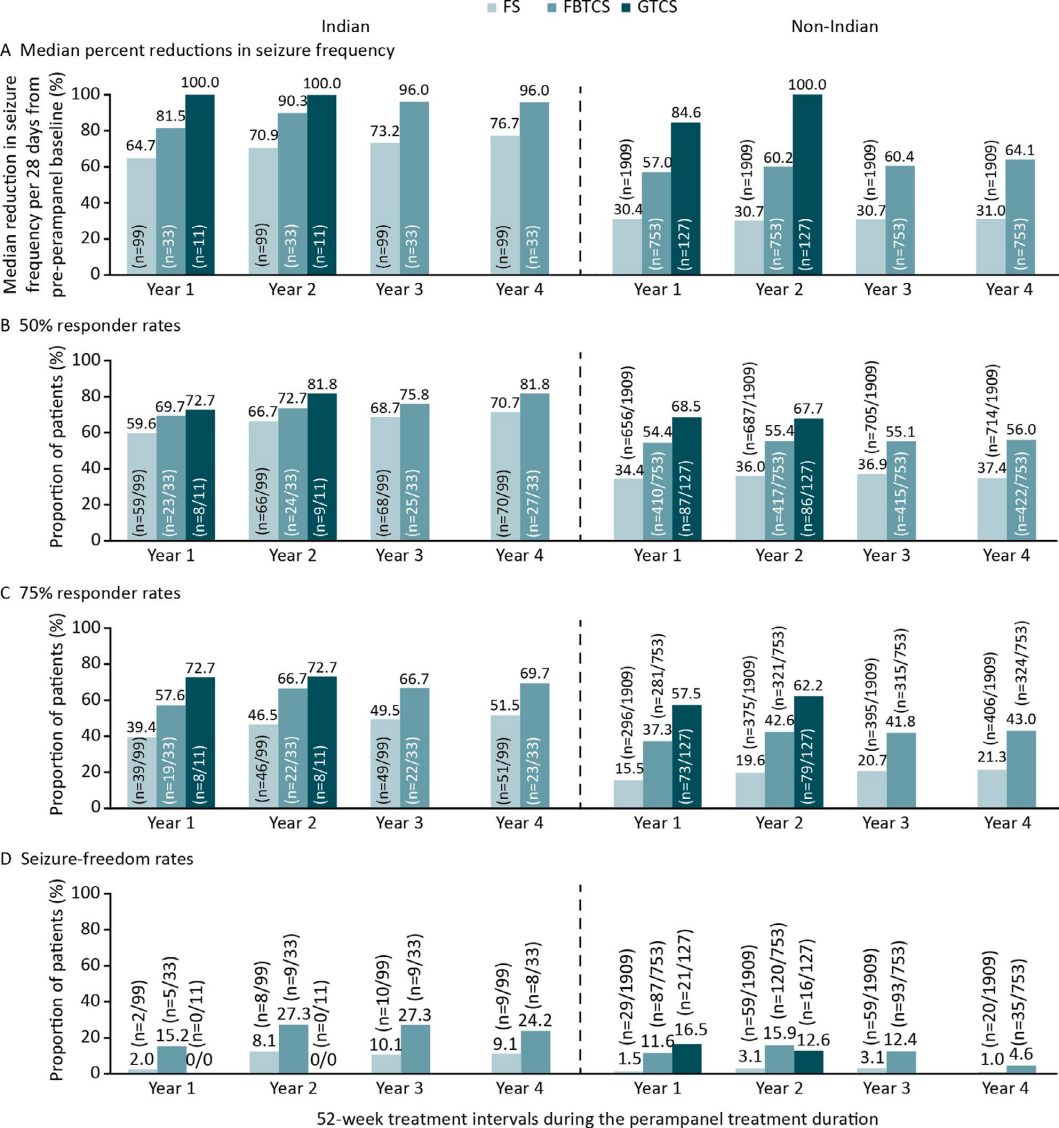
OLEx studies: A, Median percent reductions in seizure frequency per 28 days from pre‐perampanel baseline; B, 50% responder rates; C, 75% responder rates, and D, Seizure‐freedom rates during the perampanel treatment duration by 52‐week treatment intervals in Indian and non‐Indian patients (Full Analysis Set including early dropouts^a^). Abbreviations: FBTCS, focal to bilateral tonic‐clonic seizures; FS, focal seizures; GTCS, generalized tonic‐clonic seizures; OLEx, open‐label extension. ^a^Last observation carried forward: patients who completed or withdrew from the study had their last year of treatment carried forward to later time points; for patients who were treated for <1 year, their entire treatment period was carried forward to later time points

Following long‐term treatment with adjunctive perampanel (four years for FS and FBTCS; two years for GTCS) and including data for patients who dropped out of the study early, a reduction in the frequency of FS, FBTCS, and GTCS per 28 days was observed across the perampanel treatment duration in Indian and non‐Indian patients (Figure 2A). Fifty‐percent and 75% responder rates were maintained for up to four years for FS and FBTCS and up to two years for GTCS (Figures 2B,C). Seizure‐freedom rates were also maintained for up to four years for FS and FBTCS, but 0/11 Indian patients with GTCS achieved seizure freedom (Figure 2D).

### 3.6 OLEx studies: safety outcomes

TEAEs were reported in 92 (83.6%) Indian patients vs 1853 (91.0%) non‐Indian patients; 65 (59.1%) Indian and 1644 (80.7%) non‐Indian patients had treatment‐related TEAEs, respectively (Tables 2 and 3). The most common TEAEs were pyrexia, dizziness, and eosinophilia (Indian patients), and dizziness, somnolence, and headache (non‐Indian patients). Serious TEAEs were reported in 9 (8.2%) Indian patients and included convulsion (n = 3), and ankle fracture, ataxia, atrioventricular dissociation, brain contusion, carpal tunnel syndrome, drug withdrawal convulsions, grand mal convulsion, hypertrophic cardiomyopathy, status epilepticus, subdural hemorrhage, tuberculosis, typhoid fever, muscular weakness, pyrexia, and road traffic accident (n = 1 each). Two deaths were reported due to road traffic accident and subdural hemorrhage/brain contusion. Overall, 8 (7.3%) Indian patients experienced TEAEs leading to withdrawal including abnormal behavior, aggression, blood creatinine increased, decreased appetite, dizziness, hypertrophic cardiomyopathy, irritability, nausea, nervous system disorder, subdural hemorrhage, reduced visual acuity, and road traffic accident (n = 1 each). Serious TEAEs were reported in 385 (18.9%) non‐Indian patients, and there were 13 deaths; 355 (17.4%) patients experienced TEAEs leading to discontinuation.

## 4 DISCUSSION

In this post hoc analysis, once‐daily adjunctive perampanel was efficacious and well tolerated in patients with FS (with or without FBTCS) or GTCS in India. Seizure control established during the double‐blind studies was maintained for up to four years for FS and FBTCS and up to two years for GTCS during the OLEx studies.

Given previous studies of adjunctive perampanel have predominantly included Caucasian populations,^10^, ^11^, ^12^, ^13^, ^15^ it is important to assess outcomes in Asian populations.^6^ A pooled analysis in patients with FS from Studies 304, 305, 306, and 335 found no significant difference in efficacy between Asian (patients from China, Hong Kong, India, Japan, South Korea, Malaysia, Philippines, Taiwan, and Thailand) and non‐Asian (predominantly Caucasian) populations.^19^ Adjunctive perampanel 8 and 12 mg/day were consistently associated with significantly greater median percent reductions in seizure frequency (Asian: both *P* < .0001; non‐Asian: *P* < .0001 and *P* < .001, respectively) and 50% responder rates compared with placebo (Asian: both *P* < .0001; non‐Asian: *P* < .0001 and *P* < .001, respectively).^19^ Licensing of new ASMs in Asia is by individual countries,^3^ possibly due to the heterogeneous populations across Asia,^3^ so subgroup analyses from individual countries may provide important efficacy and safety data to support the use of ASMs in individual countries.

In our post hoc analysis in India, perampanel 4–12 mg/day combined was associated with greater reductions in the frequency of FS and FBTCS vs placebo, and greater 50% responder and seizure‐freedom rates. Similar responses to perampanel and placebo were generally observed in Indian vs non‐Indian patients for FS and FBTCS. However, in the Indian cohort, differences between perampanel and placebo often did not reach statistical significance, unlike in the non‐Indian cohort, which may be attributable to smaller sample sizes in the former. These results are generally consistent with those previously reported in Asian vs non‐Asian patients.^19^ However, in our analysis, the 12‐mg/day dose did not confer additional efficacy vs placebo in Indian patients, which may be due to the small patient numbers included for this group (FS, n = 14; FBTCS, n = 5). Therefore, additional analysis is required to confirm the efficacy of the 12‐mg/day dose in India. Despite this, our results suggest that Indian race does not differ in regard to the efficacy of perampanel in patients with FS, with or without FBTCS.

International Study 332 showed that perampanel 8 mg/day conferred significantly greater median percent reductions in GTCS frequency (*P* < .0001) and 50% responder rates (*P* = .0019) vs placebo.^15^ Our results are consistent with those from the full Study 332 population since perampanel 8 mg/day was associated with greater reductions in GTCS frequency and greater 50% responder and seizure‐freedom rates compared with placebo in Indian patients. These data provide evidence to suggest perampanel efficacy does not differ based on Indian race in patients with GTCS. Pharmacokinetic/pharmacodynamic analyses may be required to confirm if Indian race affects the perampanel exposure–response relationship for all seizure types.

In the current analysis, safety data were pooled for all seizure types. The safety profile in Indian patients during the double‐blind studies was generally consistent with the known safety profile of perampanel^7^, ^8^; the most common TEAEs were dizziness and headache, which have been reported as common TEAEs during previous perampanel studies in predominantly Caucasian populations.^10^, ^11^, ^12^, ^13^, ^15^ TEAE incidence during the double‐blind studies was lower for Indian patients (placebo, 53.8%; perampanel 2–12 mg/day, 58.4%) vs non‐Indian patients (placebo, 67.7%; perampanel 2–12 mg/day, 78.3%) and similar, although slightly lower, than previously reported for patients with FS in Asia‐Pacific Study 335 (placebo, 66.5%; perampanel 4–12 mg/day, 76.5%)^14^ and a pooled analysis of Studies 304, 305, and 306 (placebo, 66.5%; perampanel 2–12 mg/day, 77.0%),^20^ as well as in patients with GTCS from Study 332 (placebo, 72.0%; perampanel 8 mg/day, 82.7%).^15^ Serious TEAEs were only reported in one placebo‐treated Indian patient and two perampanel‐treated patients, and TEAEs leading to withdrawal were reported in one perampanel‐treated patient, demonstrating that adjunctive perampanel was well tolerated in patients from India.

Our analyses of OLEx data allowed assessment of long‐term effects of perampanel on seizure control. Since patients retained on treatment at later time points are likely to comprise those who tolerated and responded to perampanel, dropout analyses were conducted in the current analysis to account for potential selection bias at later treatment intervals. Across the perampanel treatment duration, improvements in seizure control were observed for up to four years in Indian patients with FS and FBTCS and two years in patients with GTCS. Perampanel was particularly effective for FBTCS and GTCS. High response rates for patients with FBTCS has previously been shown in OLEx Study 307 in patients who had ≥2 years’ perampanel exposure.^18^ The additional efficacy of perampanel against generalized seizure types may be related to its mechanism of action as a selective AMPA‐receptor antagonist.^18^, ^21^ AMPA receptors are considered a rationale target for controlling generalized seizures since they have been implicated in a number of disorders characterized by overexcitation,^21^, ^22^, ^23^ and increasing evidence suggests generalized seizures are characterized by abnormalities in cortical hyperexcitability that are affected by ASM use.^24^, ^25^

OLEx studies provide valuable information on the long‐term safety of ASMs, particularly regarding new safety signals that may not emerge during shorter clinical trials.^16^ The long‐term safety profile in India was generally consistent with non‐Indian patients, the double‐blind studies, and the known safety profile of perampanel.^7^, ^8^ Importantly, no new safety signals emerged during long‐term treatment. Although perampanel is already approved in India for adjunctive treatment of FS^9^ and GTCS in patients aged ≥ 12 years (data on file, Eisai Inc., Woodcliff Lake, NJ, USA), our results provide further evidence supporting the long‐term use of perampanel for these seizure types.

Potential limitations of this analysis include those inherent to post hoc analyses, and in particular, the use of the LOCF approach since using data from a previous assessment may not accurately reflect the efficacy of treatment for the rest of the follow‐up. In addition, the small number of patients in some treatment groups prevents a meaningful interpretation of data since statistical analyses are not robust. The open‐label nature of the OLEx studies means no placebo data are available with which to compare outcomes during long‐term treatment. Further analyses in larger groups of patients may be required to confirm our results and to provide further evidence to support the efficacy of the 12‐mg/day dose.

In conclusion, this post hoc analysis provides the first perampanel data in an Indian population of patients with epilepsy. Our results demonstrate that the short‐ and long‐term efficacy, safety, and tolerability profile of adjunctive perampanel in Indian patients with FS (with or without FBTCS) or GTCS are consistent with those reported during the global Phase III studies and provide further guidance for the use of perampanel in Indian patients with epilepsy in real‐life settings and the personalization of treatment decisions for this population.

## CONFLICTS OF INTEREST

MMM, MG, and SAJ do not have any real or apparent conflicts of interest to disclose in relation to this work. AP is an employee of Eisai Ltd. AD is an employee of Eisai Singapore Pte., Ltd. MM is an employee of Eisai Inc. We confirm that we have read the Journal's position on issues involved in ethical publication and affirm that this report is consistent with those guidelines.

## AUTHOR CONTRIBUTIONS

All authors provided substantial contributions to the conception, design of the post hoc analyses, acquisition of data, or the data analysis. All authors were involved in the interpretation of the results, the drafting, reviewing, and approval of the manuscript, and in the decision to submit the article for publication. All authors also confirm accountability for the accuracy and integrity of the work.

## Supporting information

Fig S1

Supplementary Material
