# Supplementary figures and images for: Efficacy and safety of adjunctive perampanel in patients with focal seizures or generalized tonic‐clonic seizures: Post hoc analysis of Phase II and Phase III double‐blind and open‐label extension studies in India
Source: Epilepsia Open. 2021 Feb 8;6(1):90–101. doi: 10.1002/epi4.12448 (PMC7918331; doi:10.1002/epi4.12448)

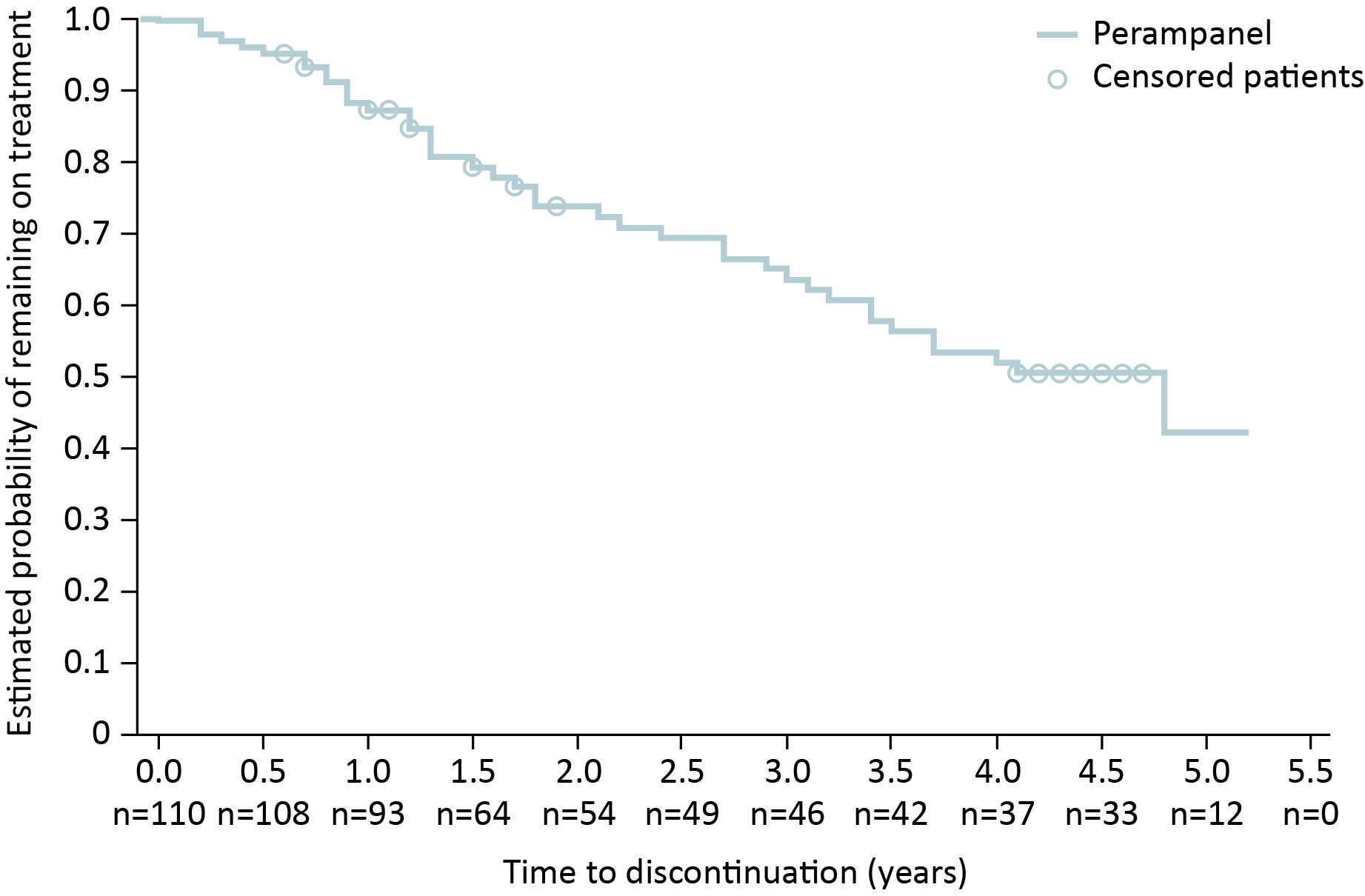

Supplement: Supplementary file 1 — Fig S1 [file EPI4-6-90-s001.tif]
