# Supplementary material for: Efficacy and safety of adjunctive perampanel in patients with focal seizures or generalized tonic‐clonic seizures: Post hoc analysis of Phase II and Phase III double‐blind and open‐label extension studies in India
Source: Epilepsia Open. 2021 Feb 8;6(1):90–101. doi: 10.1002/epi4.12448 (PMC7918331; doi:10.1002/epi4.12448)
Supplement: Supplementary file 2 — Supplementary Material [file EPI4-6-90-s002.docx]

**SUPPLEMENTARY MATERIALS**

**FIGURE S1** Kaplan–Meier plot of probability of remaining on treatment for Indian patients enrolled in the OLEx studies

**
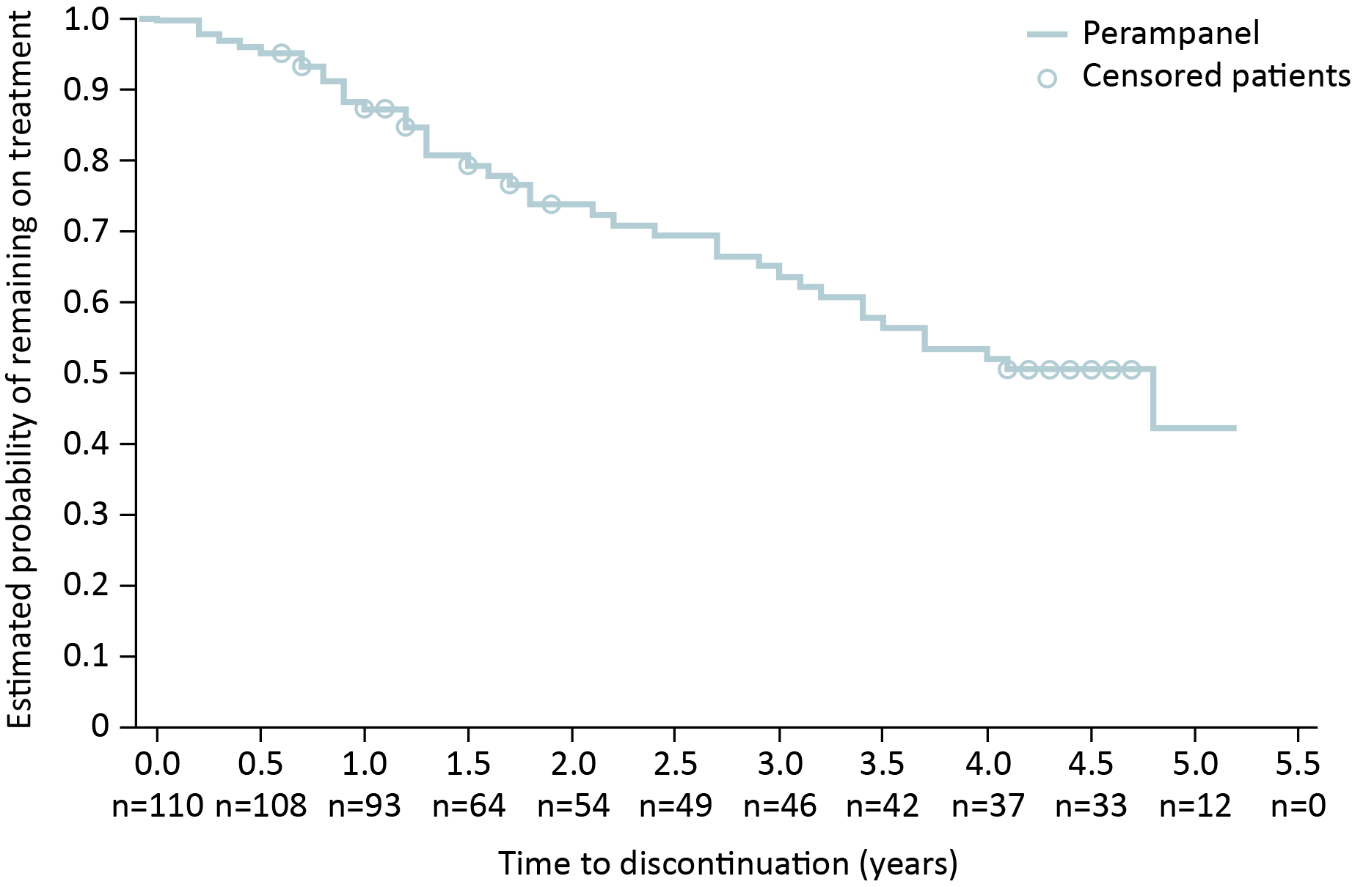
**

Abbreviation: OLEx, open-label extension.

# Patients who completed the full treatment period are included as censored events.

# TABLE S1 Double-blind studies: median (95% CI) difference between perampanel and placebo for percent reductions in seizure frequency per 28 days from baseline for Indian and non-Indian patients (Full Analysis Set)

| **Median (95% CI) difference from placebo** | **Perampanel dose (mg/day)** | | | | |
| --- | --- | --- | --- | --- | --- |
|  | **2** | **4** | **8** | **12** | **4–12** |
| **Indian patients** |  |  |  |  |  |
| **FS** | 4.3 (-30.7, 34.4) | 27.5 (-18.8, 71.3) | -19.4 (-39.8, 7.4) | 5.6 (-24.6, 42.1) | -7.3 (-30.0, 15.2) |
| **FBTCS** | 115.9 (NC, NC) | 35.9 (-54.2, 750.6) | -30.9 (-66.0, 0.0) | 33.9 (-30.4, 92.5) | 6.8 (-49.4, 39.7) |
| **GTCS** | – | – | 53.8 (-133.9, 41.0) | – | – |
| **Non-Indian patients** |  |  |  |  |  |
| **FS** | -2.1 (-9.9, 5.7) | -9.3 (-15.3, -3.4) | -18.4 (-23.7, -13.2) | -20.3 (-25.9, -14.6) | -16.8 (-21.1, -12.4) |
| **FBTCS** | -14.7 (-33.2, 1.6) | -20.1 (-33.5, -6.6) | -36.5 (-48.2, -25.1) | -33.6 (-46.2, -21.8) | -31.4 (-41.4, -21.9) |
| **GTCS** | – | – | -28.4 (-43.9, -14.1) | – | – |

Abbreviations: CI, confidence interval; FBTCS, focal to bilateral tonic-clonic seizure; FS, focal seizure; GTCS, generalized tonic-clonic seizure; NC, not calculated.

Positive values indicate a greater reduction in seizure frequency compared with placebo.
